# Increased expression of myelin-associated genes in frontal cortex of *SNCA* overexpressing rats and Parkinson’s disease patients

**DOI:** 10.18632/aging.103935

**Published:** 2020-10-05

**Authors:** Thomas Hentrich, Zinah Wassouf, Christine Ehrhardt, Eva Haas, James D. Mills, Eleonora Aronica, Tiago Fleming Outeiro, Jeannette Hübener-Schmid, Olaf Riess, Nicolas Casadei, Julia M. Schulze-Hentrich

**Affiliations:** 1 Institute of Medical Genetics and Applied Genomics, University of Tübingen, Tübingen 72076, Germany; 2 German Center for Neurodegenerative Diseases, Göttingen 37073, Germany; 3 Department of Experimental Neurodegeneration, Center for Biostructural Imaging of Neurodegeneration, University Medical Center Göttingen, Göttingen 37073, Germany; 4 Department of (Neuro) Pathology, Amsterdam UMC, University of Amsterdam, Amsterdam Neuroscience, Amsterdam 1105 AZ, The Netherlands; 5 Max Planck Institute for Experimental Medicine, Göttingen 37073, Germany; 6 Translational and Clinical Research Institute, Faculty of Medical Sciences, Newcastle University, Framlington Place, Newcastle Upon Tyne NE2 4HH, UK; 7 DFG NGS Competence Center Tübingen, Tübingen 72076, Germany

**Keywords:** Parkinson’s disease, frontal cortex, myelination, oligodendrocytes, transcriptome analysis

## Abstract

Parkinson’s disease (PD) is an age-dependent neurodegenerative disorder. Besides characteristic motor symptoms, patients suffer from cognitive impairments linked to pathology in cortical areas. Due to obvious challenges in tracing the underlying molecular perturbations in human brain over time, we took advantage of a well-characterized PD rat model. Using RNA sequencing, we profiled the frontocortical transcriptome of post-mortem patient samples and aligned expression changes with perturbation patterns obtained in the model at 5 and 12 months of age reflecting a presymptomatic and symptomatic time point. Integrating cell type-specific reference data, we identified a shared expression signature between both species that pointed to oligodendrocyte-specific, myelin-associated genes. Drawing on longitudinal information from the model, their nearly identical upregulation in both species could be traced to two distinctive perturbance modes. While one mode exhibited age-independent alterations that affected genes including proteolipid protein 1 (*PLP1*), the other mode, impacting on genes like myelin-associated glycoprotein (*MAG*), was characterized by interferences of disease gene and adequate expression adaptations along aging. Our results highlight that even for a group of functionally linked genes distinct interference mechanisms may underlie disease progression that cannot be distinguished by examining the terminal point alone but instead require longitudinal interrogation of the system.

## INTRODUCTION

Parkinson’s disease (PD) is an age-related neurodegenerative disease characterized by increasing accumulation of alpha-synuclein, encoded by the *SNCA* gene [1]. Clinical motor symptoms of PD such as tremor, rigidity, and bradykinesia are linked to cell loss in the substantia nigra pars compacta. Additionally, an array of cognitive deficits, including impairment of executive function, language, visuospatial/visuoconstructive abilities, memory, depression, apathy, and impulse control disorders are also observed, even in early stages of PD [2–6]. These non-motor symptoms have been associated with disturbances of information flow through frontal-subcortical networks [7]. Through interactions with basal ganglia, the frontal cortex is also involved in controlling motor functions [7]. In line, damage to the frontal cortex or basal ganglia lead to several comparable cognitive impairments [8–11]. Hence, investigating disease-associated changes in frontal cortex may aid in understanding the manifestation of PD.

Through positron emission tomography (PET), functional MRI (fMRI), and magnetic resonance spectroscopy (MRS) functional and metabolic disturbances have been identified in frontal cortex of PD patients [12]. In addition, transcriptomic and proteomic investigations describe abnormalities in mitochondrial, protein folding, and ubiquitin conjugation pathways in the prefrontal cortex of PD patients [13, 14]. However, these studies remain limited in explaining how these molecular changes develop as PD progresses.

In order to trace these molecular perturbations to deviating gene activities in early stages of PD, we used a transgenic rat model of PD overexpressing the full-length human wildtype *SNCA* locus on a BAC/PAC fusion construct [15]. In this model, overexpression of *SNCA* leads to C-terminal truncation and conversion into insoluble, proteinase K–resistant alpha-synuclein species, strongly enriched in nigrostriatal system. The progressive aggregation of alpha-synuclein results in age-dependent loss of dopaminergic cells and motor deficits surfacing at around 12 months of age. Thus, we analysed the frontocortical transcriptome of 5- and 12-month-old animals using RNA sequencing (RNA-seq) to capture a presymptomatic and symptomatic time point. In parallel, we profiled the frontocortical transcriptome in PD patients. Through integration of results across rat and human with cell–type–specific data, we revealed a distinctive increase of expression in myelin-associated genes attributed to oligodendrocytes that was shared between both organisms. Taking advantage of the temporal dimension in the rat data, we traced these perturbations to originate from either genotype or age-genotype effects.

## RESULTS

### *SNCA* overexpression causes age-dependent gene expression changes in frontocortical rat tissue

Animal models circumvent the inaccessibility that largely exists for patient brain tissue prior to death and, hence, are ideal for studying pathogenic events that unfold over time. The well-characterized transgenic rat model we used in this study overexpresses full-length human *SNCA* and recapitulates typical PD features such as alpha-synuclein aggregation, progressive death of dopaminergic cells with associated motor symptoms, and neuroinflammation [15–17]. To identify gene expression changes over time, we chose the 5- and 12-month-old time point that reflect a presymptomatic and symptomatic stage in the model at adulthood. Using RNA-seq, we profiled the transcriptome of frontal cortex in five transgenic (TG) and five wildtype (WT) rats at both time points (Supplementary Figure 1) and determined differentially expressed genes (DEGs) along the genotype and age axis.

**Figure 1:**
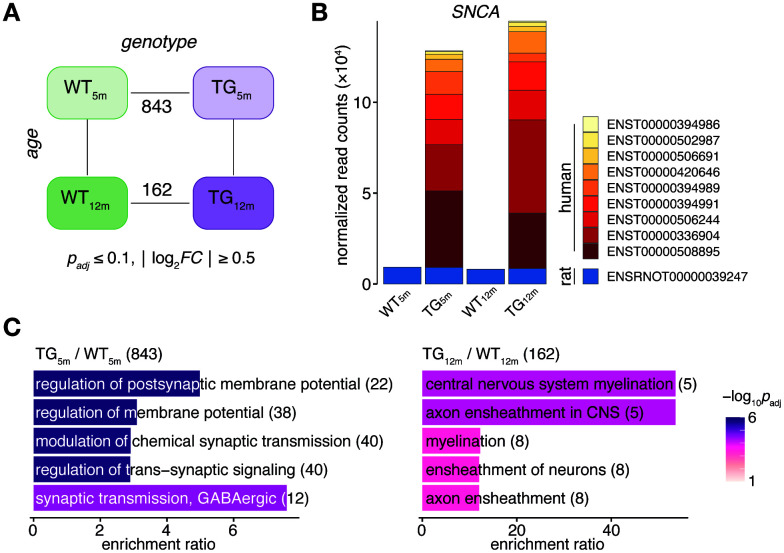
***SNCA* overexpression in rat causes age-dependent gene expression perturbations in frontocortical tissue.** (**A**) Schematic diagram of experimental groups along the genotype (wild-type WT, transgenic TG), and age (5 and 12 months) axis highlighting number of differentially expressed genes (DEGs) between TG and WT rats using indicated significance cut-offs. (**B**) Composition and relative expression levels of rat and human *SNCA* transcript isoforms across experimental groups. (**C**) Overrepresented biological processes among 843 DEGs identified in 5-month-old (left panel) and among 162 DEGs in 12-month-old TG rats (right panel). Top five significant terms, their adjusted *p*-values, enrichment ratios, and DEG count shown.

With respect to genotype, 843 DEGs (553 up- and 290 downregulated) in 5-month-old and 162 DEGs (108 up- and 54 downregulated) in 12-month-old rats were identified (Figure 1A). While the endogenous *SNCA* transcript showed stable expression across experimental groups, transcripts from the transgene locus led to a >10-fold overexpression of SNCA in TG animals (Figure 1B). The *SNCA* expression level and transcript shares remained largely similar for both time points (Figure 1B). Functionally, the 843 DEGs identified in 5-month-old animals were enriched for biological processes linked to synaptic functions including regulation of postsynaptic membrane potential and modulation of chemical synaptic transmission (Figure 1C). The 162 DEGs identified in 12-month-old animals were attributed to processes linked to myelination such as central nervous system myelination and axon ensheathment in central nervous system (Figure 1C).

The different DEG counts observed at 5 and 12 months of age despite a stable overexpression of *SNCA* imply interferences between the transgene and the host system that changed during this time window.

### Gene expression patterns point to interactions between *SNCA* overexpression and age-related adaptations of gene activity

**Figure 2:**
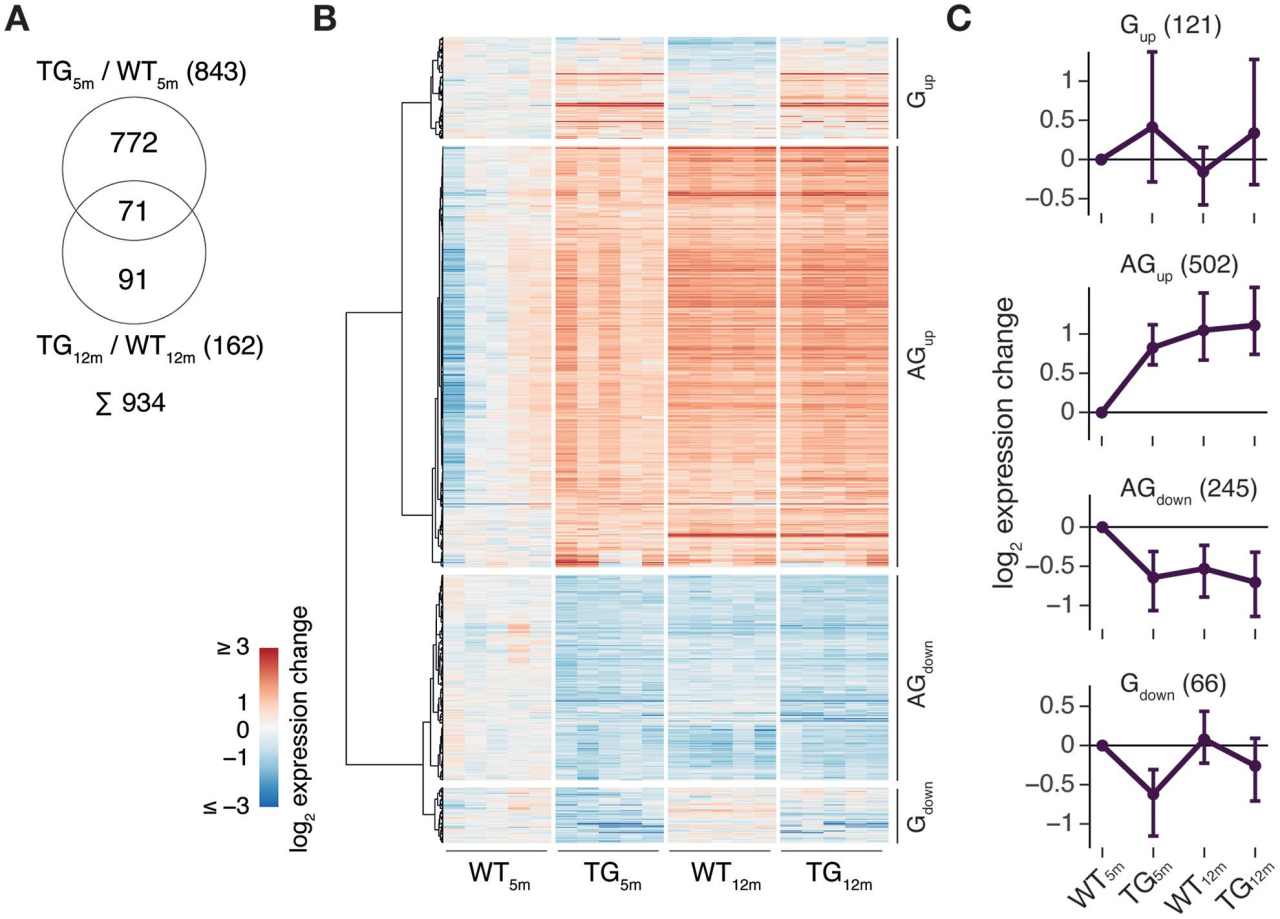
**Gene expression patterns point to interactions between *SNCA* overexpression and age-related adaptations in gene activity.** (**A**) Venn diagram comparing DEGs identified in 5- and 12-month-old TG rats. (**B**) Hierarchically clustered expression changes (relative to WT_5m_) for all 934 DEGs identified in 5- and 12-month-old TG rats partitioned into four main perturbance patterns. (**C**) Gene clusters (see B) summarized as expression medoids (±SD). Cluster cardinalities indicated in brackets.

Against a presumed increase of gene expression perturbations over time given an age-dependent disease, DEGs dropped from 843 to 162 between 5 and 12 months of age with only 71 DEGs overlapping (Figure 2A). To better understand this observation, DEGs for both time points were visualized across experimental groups and showed perturbance patterns that partition into four major clusters (Figure 2B). Summarized as medoids, two classes of nearly mirror-imaged expression profiles, G (abbr: Genotype) and AG (abbr: Age-Genotype), became apparent (Figure 2C). Class G contained genes that were either up- or downregulated at both time points and, hence, reflected a mainly genotype-driven expression change irrespective of age (Figure 2B, 2C). In contrast, genes in class AG show differential expression in 5-month-old TG rats only. These DEGs were no longer identified at the 12-month time point, as WT animals underwent expression adaptations during that time which are strikingly similar to the expression difference observed in the 5-month-old animals (Figure 2B, 2C). Hence, class AG suggests age-dependent expression perturbation for certain genes.

### Distinct classes of aberrant age-related adaptations in rats overexpressing SNC

**Figure 3:**
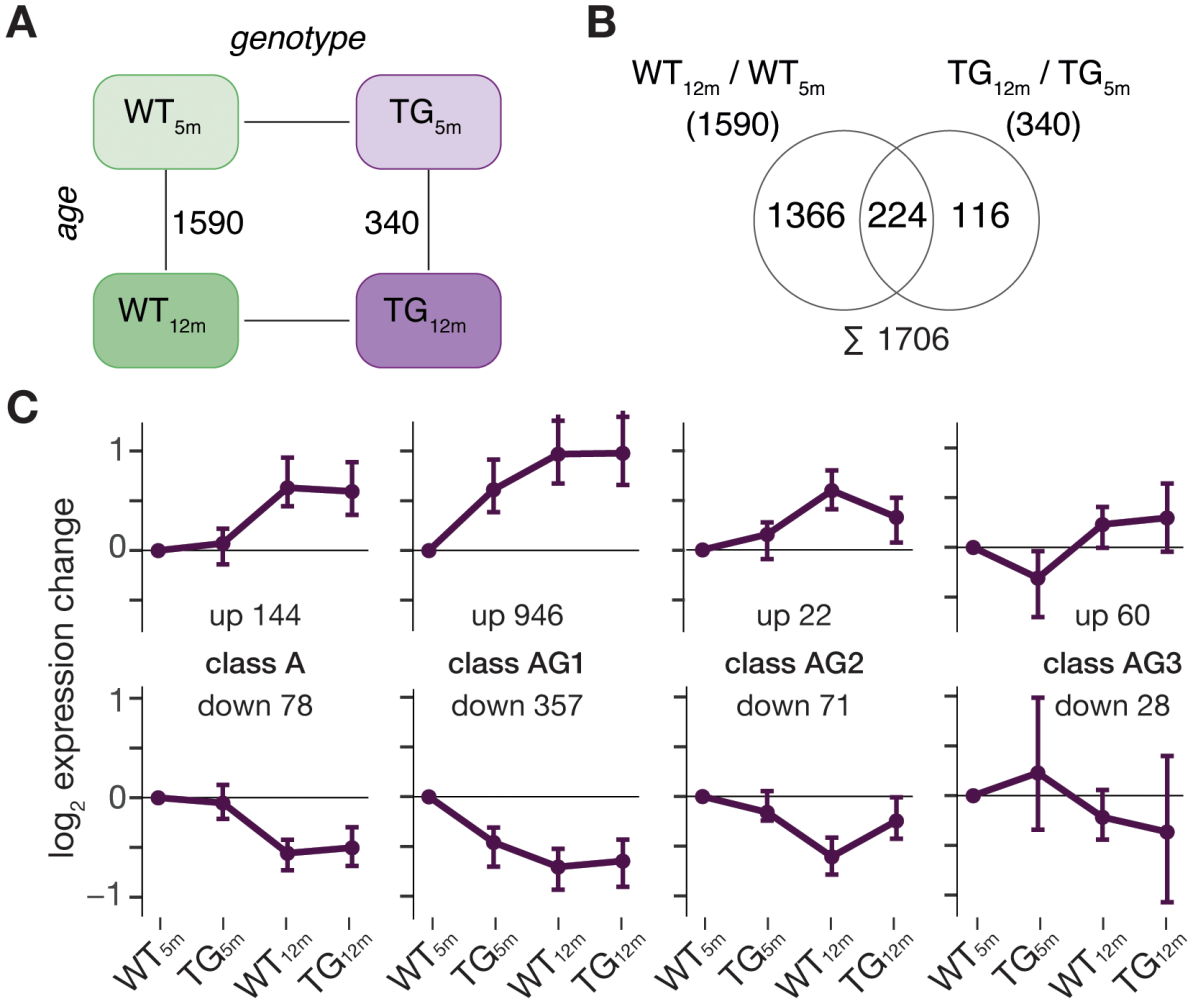
**Age-related adaptations in frontocortical gene expression are perturbed in context of *SNCA* overexpression.** (**A**) Schematic diagram of experimental groups highlighting differentially expressed genes between 5- and 12-month-old rats using the same significance cut-offs of *p*_adj_ ≤ 0.1 and │log_2_FC│≥ 0.5 as above. (**B**) Venn diagram comparing DEGs identified in WT and TG rats between 5 and 12 months of age. (**C**) Partitioning of 1706 DEGs based on their gene expression in 5- and 12-month-old WT and TG rats. Subplots show expression medoids (± SD) of eight primary gene clusters grouped into four classes. Cluster cardinalities indicated.

Consequently, a proper understanding of transgene-induced perturbations requires putting them into perspective against typical adaptations of gene activity over time. Therefore, differential expression was determined along the age dimension among experimental groups. A total of 1590 (1094 up- and 496 downregulated) and 340 DEGs (277 up- and 63 downregulated) were identified for WT and TG animals, respectively (Figure 3A). The different DEG counts and their incomplete overlap (Figure 3B) suggest age-dependent adaptations of gene activity occurred differently in WT and TG animals. Partitioning these DEGs according to their expression patterns over time (Supplementary Figure 2) led to four primary classes each comprising two nearly mirror-imaged profiles (Figure 3C). While there were still some genes, whose expression adaptations in TG animals corresponded to the WT condition (class A, abbr: Age), the majority of genes fell into age-genotype-dependent classes AG1, AG2, and AG3 that clearly showed aberrant expression adaptations in TG animals (Figure 3C). Specifically, classes AG1 and AG3 (similar to class AG, Figure 2B, 2C) showed differential expression in 5- but not in 12-month-old TG animals (Figure 3C). Interestingly, class AG2 contained genes that showed expression adaptations in WT animals only, which TG animals failed to undergo (Figure 3C). This adaptation failure in context of *SNCA* overexpression was in line with comparable observations we made in a mouse model overexpressing human *SNCA* [18].

Together, these results point at distinct classes of aberrant age-related adaptations in transgenic rats, which only become apparent when taking several time points over disease progression into account.

### Shared expression changes in rats overexpressing *SNCA* and PD patients point to myelin-linked genes attributed to oligodendrocytes.

**Figure 4:**
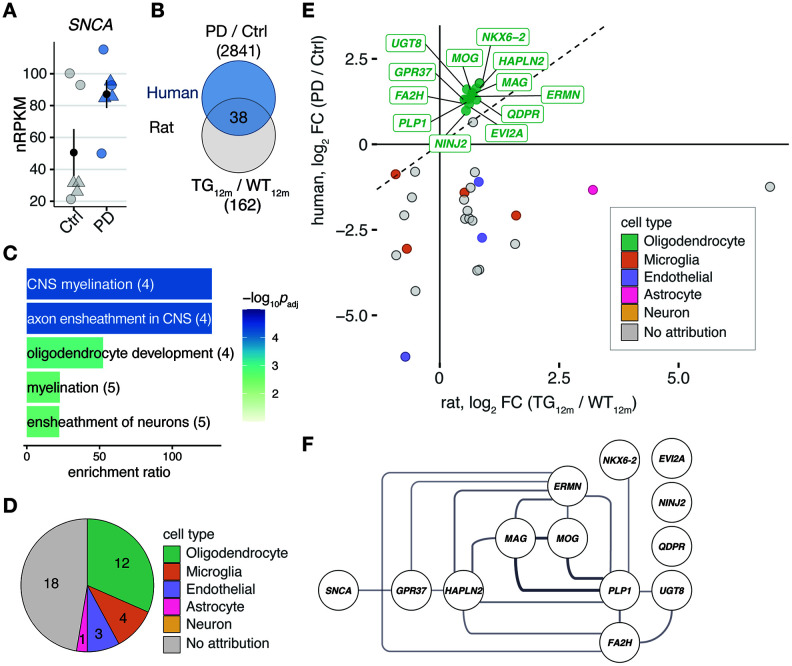
**Shared differentially expressed genes in the rat model overexpressing *SNCA* and PD patients point to myelination and oligodendrocytes.** (**A**) Expression changes of *SNCA* in PD patients compared to controls plotted as individual data points with mean ± SEM. Circles represent females, rectangles males. (**B**) Venn diagram comparing 162 DEGs identified in 12-month-old TG rats and 2841 DEGs identified in frontal cortex of PD patients according to cut-offs of *p*_adj_ ≤ 0.1 and │log_2_FC│≥ 0.5. (**C**) Overrepresented biological processes among 38 DEGs shared between rat and human (see **B**). Five most significant terms, their adjusted *p*-values, enrichment ratios, and underlying gene count shown. (**D**) Pie chart showing attribution of 38 DEGs shared between rat and human to cell types according to reference data from McKenzie et al. [19]. (**E**) Scatter plot of 38 DEGs identified in frontal cortex of rat and human. Cell type attributions color-coded. Oligodendrocyte DEGs labelled. (**F**) Protein-protein interaction network derived from 38 DEGs attributed to oligodendrocytes plus *SNCA*. Interactions according to String database. Only connected nodes shown. Line width reflects String interaction score.

In order to relate gene expression changes between rat and human, we profiled the transcriptome of post mortem tissue from frontal cortex of six PD patients and six healthy controls using RNA-seq (see Supplementary Table 1). *SNCA* itself showed a tendency of upregulation (log_2_FC 0.79, *p*_adj_ 0.18) in PD patients (Figure 4A). Among the 2841 DEGs (689 up- and 2152 downregulated) differentially expressed in patients, 38 orthologues were identified in 12-month-old rats, too (Figure 4B). These shared DEGs were most significantly enriched for the biological processes central nervous system myelination, axon ensheathment in central nervous system, and oligodendrocyte development (Figure 4C).

**Figure 5:**
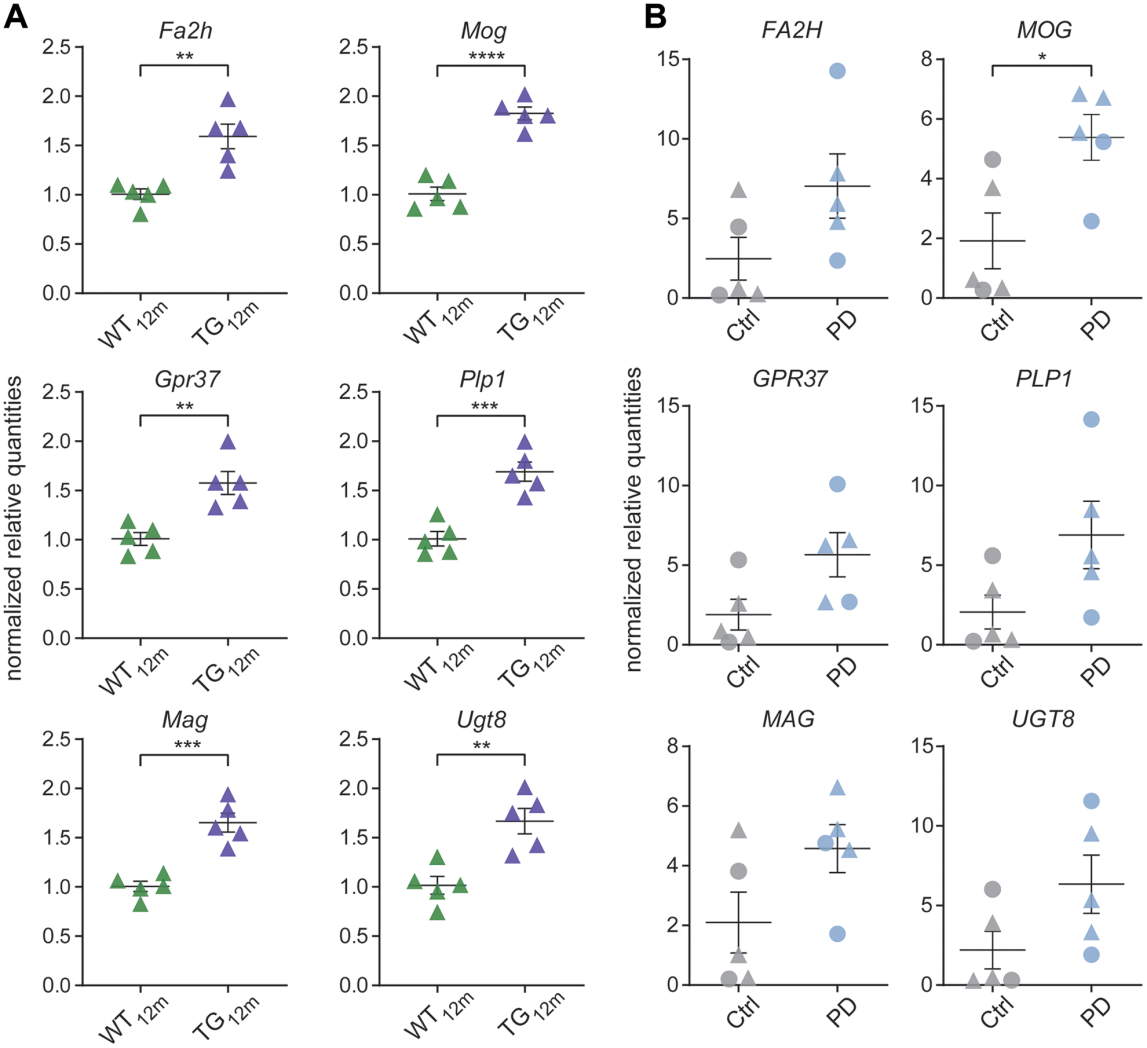
**Validation of oligodendrocyte-associated gene expression changes using RT-qPCR.** (**A**) RNA-seq results of shared oligodendrocyte-specific targets were verified by RT-qPCR in rats. RT-qPCR normalized quantities shown relative to WT with individual data points plotted with mean ± SEM. Significances based on unpaired two-tailed *t*-tests with *p < 0.05, **p < 0.01, ***p < 0.001, ****p < 0.0001. (**B**) RNA-seq results of shared oligodendrocyte-specific targets were verified by RT-qPCR in human. RT-qPCR normalized quantities shown relative to controls with individual data points plotted with mean ± SEM. Circles represent females, rectangles males. Significances based on unpaired two-tailed *t*-tests with *p < 0.05.

To further explore the link to oligodendrocytes, the 38 DEGs were examined for their cell type-specific expression in human against reference data for neurons, astrocytes, microglia, oligodendrocytes, and endothelial cells [19]. Strikingly, DEGs specifically expressed in oligodendrocytes represented the largest group (Figure 4D) and showed nearly identical upregulation in 12-month-old rats and PD patients (Figure 4E). Including *SNCA* to the oligodendrocyte-specific genes through the network of direct (physical) or indirect (functional) protein-protein interactions obtained from String [20], connected SNCA to GPR37 (Figure 4F), a G protein-coupled receptor which has previously been associated with PD [21, 22]. To validate these RNA-seq findings, RT-qPCR was performed for six of the shared oligodendrocyte-linked genes: *Fa2h*, *Gpr37*, *Mag*, *Mog*, *Plp1*, and *Ugt8* (Figure 5). In rats, a significant increase in gene expression was observed for all six genes (Figure 5A). In humans, the increase of *MOG* in PD patients reached significance and all other genes showed a strong upregulation trend (Figure 5B).

Together, these results suggest oligodendrocyte-specific perturbances in frontocortical gene expression shared between the rat model and human patients, which potentially impacts on the interaction network of myelin-associated proteins.

### Genotype- and age-related perturbations in gene activity cause increase of myelin-linked genes in rats overexpressing SNCA and PD patients

**Figure 6:**
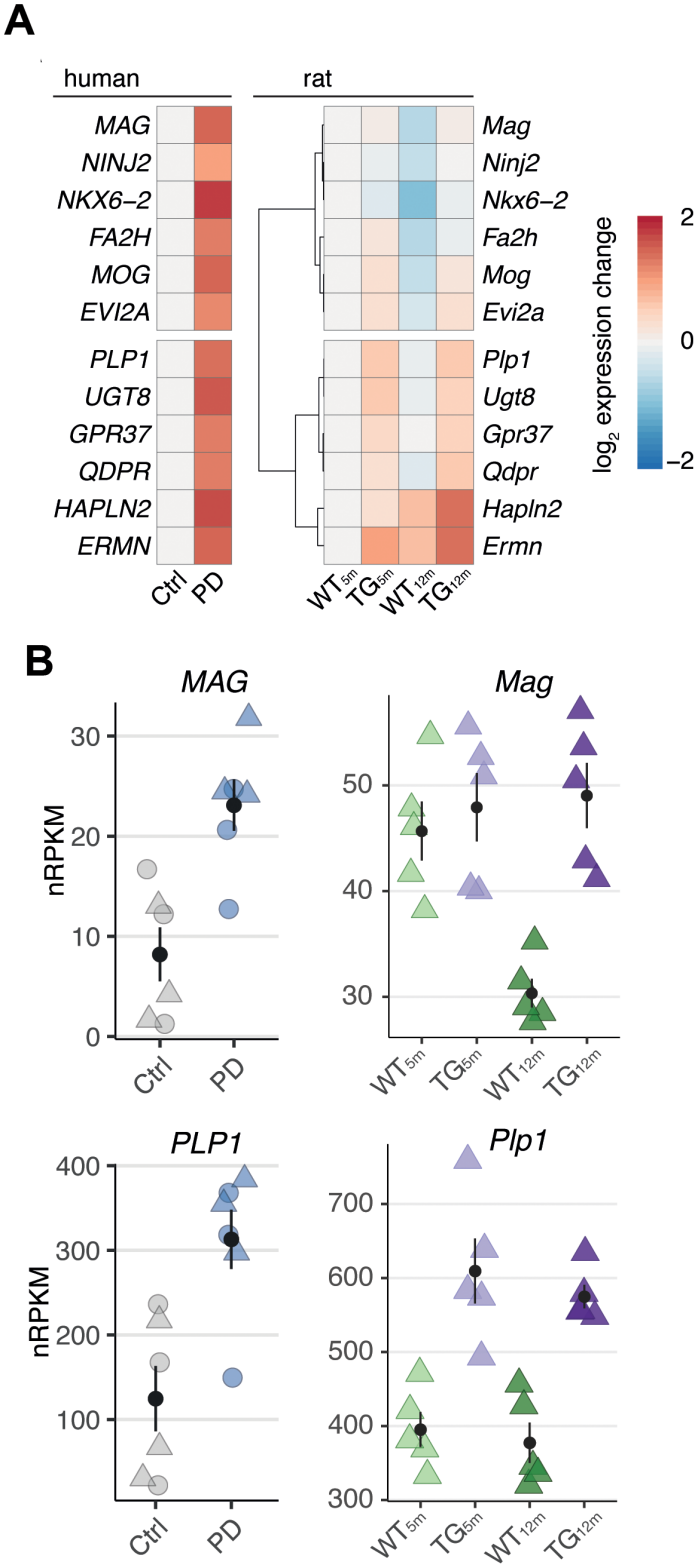
**Genotype- and age-related perturbations in gene activity cause increase of myelin-linked genes in rats overexpressing *SNCA* and PD patients.** (**A**) Left panel shows heatmap of expression changes in frontal cortex of PD patients relative to healthy controls for shared DEGs attributed to oligodendrocytes. Right panel shows hierarchically clustered rat expression changes (relative to WT_5m_) for the same DEGs. (**B**) Rat and human expression changes of *PLP1* and *MAG* across experimental groups plotted as individual data points with mean ± SEM. For human data, circles represent females, rectangles males.

RNA-seq and quantitative PCR results in PD patients showed a significant increase of myelin-associated genes also identified in the rat model (Figures 5 and 6A). Due to the limitations mentioned above for human, tracing these perturbations to an earlier time point and understanding as to how they developed during disease unfolding remains challenging. Here, animal models offer unique advantages and represent ideal systems that allow staging of cohorts, standardizing environmental conditions, and obtaining time series data.

Accordingly, we examined the twelve DEGs also in rat by visualizing their expression pattern over time and noticed distinct modes of genotype- and age-related gene expression changes underlying the observed increase (Figure 6A). While, on one hand, genes like *PLP1* showed primarily genotype-driven perturbations of altered expression in TG animals irrespective of age (Figure 6B and Supplementary Figure 3) as in class G (Figure 2B, 2C), genes like *MAG* rather reflected failed age-adequate adaptations (Figure 6B and Supplementary Figure 3) as in class AG2 (Figure 3C).

Taken together, the cross-species data integration suggests oligodendrocyte-specific, myelin-associated gene expression changes to result from two distinctive perturbation modes: either age-independent effects or interferences of *SNCA* overexpression with age-adequate adaptations of the system.

## DISCUSSION

In this study, we examined transcriptome perturbations in frontal cortex of an *SNCA* overexpressing rat model and post- mortem samples of PD patients. Analyses of these data revealed a strikingly similar increased expression of myelin-associated genes shared between model and human. The temporal expression pattern of these genes in rat suggest their increase to result, on one hand, from age-independent effects of *SNCA* overexpression and, on the other hand, from interferences of the overexpressed disease gene with age-adequate adaptations in gene activity.

By drawing on cell type-specific human expression data, we were able to attribute the majority of shared differentially expressed genes in frontal cortex of the rat model and patients to be oligodendrocyte-specific. While it is already known for this cell type to play a crucial role in multiple system atrophy (MSA), another synucleinopathy characterized by overexpressed *SNCA* and accumulation of alpha-synuclein in oligodendrocytes as well as neurons [23], little is known about the role of this cell type in PD. It has been described that oligodendrocytes in PD brains may contain inclusions of alpha-synuclein, referred to as coiled bodies [24, 25].

In line, a recent study integrating GWAS results with single-cell transcriptomic data points to an important role of oligodendrocytes in PD [26]. Specifically, the study shows differentially expressed genes in post-mortem PD brains from six separate cohorts to be enriched for specific cell types: While downregulated genes are attributed to dopaminergic neurons, upregulated genes seem specific for cell types from the oligodendrocyte lineage, agreeing with our findings. In addition, upregulated genes are already strongly enriched for oligodendrocytes at Braak Stages 1-2 [26]. Consistently, we here observed in the rat model increased expression of oligodendrocyte-specific genes already at 12 months of age when motor symptoms begin to fully surface. This suggests perturbations of oligodendrocytes to precede appearance of motor symptoms and pathological changes in the substantia nigra.

A central function of oligodendrocytes is to generate myelin. Despite myelination being crucial for cells that connect cortical and subcortical brain areas [27–29], little is known about the role of myelin in context of aging and neurodegenerative processes [30, 31]. For PD in particular, which has largely been considered a grey matter disease, the role of myelin still is under active debate in the field. While reports generally agree that changes occur in white matter structure as the disease progresses [32–35], they contradict with respect to presumed consequences. On one hand, some studies interpret microstructural changes that are typical for neurodegeneration to reflect loss of white matter due to demyelination and axonal damage [36–39]. On the other hand, recent evidence suggests that changes in white matter microstructure might be compensatory [38, 40, 41].

A lower myelin water fraction, a surrogate for myelin content, has been associated with older age and suggest reduction of myelin with age [42]. Intriguingly, PD patients have a slower decline rate of the myelin water fraction with increasing age [42]. So far, the mechanisms underlying these compensatory alterations have remained unclear. It has been speculated that either the density of axonal packing may be increased in PD patients [38] or, alternatively, compensatory changes may encompass increases in myelin [42].

In line with the latter, we here observed in rat a subset of myelin-associated genes failing to undergo age-adequate adaptations so that their expression remains higher than normal at older age. Hence, failure in adaption of gene activity could indeed reflect compensatory attempts of the system to cope with the overload of disease protein. The slower reduction of myelin and myelin sheath with age in PD patients raises intriguing questions concerning the role of myelin in compensating disease-associated cognitive decline. One could speculate the disease-associated reduction in neuronal function, in turn, to be potentially counter-balanced by myelinated neurons to conduct information faster in the remaining neuronal circuit. To resolve these questions of failure or compensation with respect to the observed effects in PD, our study emphasizes that it is not sufficient to consider the end point of the disease, but to longitudinally trace and relate any alleged perturbations to adequate adaptations of gene activity essential for natural aging.

## MATERIALS AND METHODS

### Experimental animals and tissue preparation for RNA isolation

Male homozygous transgenic rats overexpressing the full-length human *SNCA* gene including its regulatory elements [15] and WT rats with the same genetic background (Sprague-Dawley) were housed in a standard environment till the age of 5 and 12 months. Experimental animals were obtained by crossing heterozygous male with heterozygous female rats, and were confirmed as homozygous or WT by genotyping with quantitative PCR using DNA from ear biopsies with primer sequences specifically for human *SNCA* (F: 5′-ccgctcgagcggtaggaccgcttgttttagac-3′; R: 5′-ctctttccacgccactatc-3′) and normalized to β-actin as reference (F: 5′-agccatgtacgtagccatcca-3′; R: 5′-tctccggagtccatcacaatg-3′). Animals were anaesthetized, decapitated, and the brain immediately dissected on ice and snap frozen in liquid nitrogen. Subsequently, the tissue was stabilized with RNAlater (Qiagen) at 4°C.

### Human samples

Human brain samples were obtained from The Netherlands Brain Bank (NBB), Netherlands Institute for Neuroscience, Amsterdam (open access: http://www.brainbank.nl). All material has been collected from donors for or from whom a written informed consent for a brain autopsy and the use of the material and clinical information for research purposes had been obtained by the NBB. Control brain tissue material was obtained at autopsy from ALS patients at the department of (Neuro)pathology of the Amsterdam UMC, Academic Medical Center, University of Amsterdam, the Netherlands. Informed consent was obtained for the use of brain tissue for research purposes.

The selected cases were than reassessed by two neuropathologists using the routine updated protocol for neurodegenerative diseases [43, 44]. We excluded cases with co-existing other neuropathological lesions (i.e. microvascular infarcts, hippocampal sclerosis, significant Alzheimer Disease neuropathologic changes) to avoid confounding factors. The presence of neuropathological PD hallmarks was assessed following consensus criteria for diagnosis of PD [45].

### RNA sequencing of rat and human frontocortical tissue

The polyadenylated fraction of RNA isolated from frontal cortex (n = 5 animals in each of the four experimental groups) was used for RNA-seq. Total RNA, miRNA, and DNA were simultaneously extracted using the DNA/RNA/microRNA Universal Kit (Qiagen) using the manufacturer protocol. Quality was assessed with an Agilent 2100 Bioanalyzer. Samples with high RNA integrity number (RIN > 7) were selected for library construction. Using the TruSeq RNA Sample Prep Kit (Illumina) and 100 ng of total RNA for each sequencing library, poly(A) selected paired-end sequencing libraries (125 bp read length) were generated according to manufacturer’s instructions. All libraries were sequenced on an Illumina HiSeq 2500 platform at a depth of 20–35 million reads each.

Human samples presented a quality with RNA integrity number (4.8 < RIN < 7.6). Library preparation using enrichment of coding RNA was performed for 40 ng of total RNA with the TruSeq RNA Access Library Perp Kit (Illumina). Libraries were sequenced as paired-end 75 bp reads on a NextSeq 500 (Illumina) at a depth of approximately 20 – 45 million reads each.

### Quality control, alignment, differential expression, enrichment tests, and functional analyses

Read quality of RNA-seq data in fastq files was assessed using *FastQC* (v0.11.4) [46] to identify sequencing cycles with low average quality, adapter contamination, or repetitive sequences from PCR amplification. Reads were aligned using *STAR* (v2.6.0a and v2.7.0a) [47] allowing gapped alignments to account for splicing against a custom-built genome composed of the *Ensembl* R. norvegicus genome v93 and the human *SNCA* transgene as well as H. sapiens genome v95. Alignment quality was analyzed using *samtools* (v1.1) [48]. Normalized read counts for all genes were obtained using *Rsubread* (v2.0.0) and *DESeq2* (v1.18.1) [49]. Transcripts covered with less than 50 reads were excluded from subsequent analyses leaving 12,307 (rat) and 15,337 (human) genes for determining differential expression. The factorial design of the experiment was captured in a general linearized model defining gene expression as a function of genotype, age, and interaction of both. Significance thresholds were set to |log_2_ FC| ≥ 0.5 and BH-adjusted *p*-value ≤ 0.1. Surrogate variable analysis (*sva*, v3.26.0) [50] was used to minimize unwanted variation between samples. Gene-level abundances were derived from *DESeq2* as normalized read counts and used for calculating the log_2_-transformed expression changes of the expression heatmap and medoids. Ratios relative to mean expression in WT_5m_. Read counts provided by *DESeq2* also went into calculating nRPKMs (normalized Reads Per Kilobase per Million total reads) as a measure of relative gene expression as motivated before [51]. Orthologous genes between rat and human were determined with the *biomaRt* package. Cell type-specific expression data were adapted from McKenzie et al. [19] using the top ranked specificity genes based on human data. *WebGestalt* was employed to identify overrepresented biological processes among *Gene Ontology* terms [52].

### Reverse transcription-quantitative PCR (RT-qPCR)

RNA-seq results for *Fa2h*, *Gpr37*, *Mag*, *Mog*, *Plp1*, and *Ugt8* were validated using RT-qPCR with primers specific for rat and human (see Supplementary Table 2 for primers sequences). One hundred nanograms of total RNA were used for reverse transcription (*QuantiTect Reverse Transcription kit*, Qiagen) following the manufacturer’s instructions. After diluting the resulting cDNA (1:20), 2 μl were used together with *SYBR green* master mix (Qiagen) and primers (0.25 μM). Expression was calculated relative to the mean of the WT (rat) and control group (human) according to the Pfaffl model [53] after normalizing to the geometric mean relative expression of *Pgk1* and *Pdhb* reference genes in rat, and *PGK1* and *SDHA* in human. Reference genes were selected based on expression stability calculated with Genorm [54] and Normfinder [55]. Differences between the TG and WT group and well as PD patients and controls were tested with unpaired two-tailed *t*-tests using significance thresholds of *p* < 0.05.

### Data availability

Human RNA-seq data have been deposited at the European Genome-phenome Archive (EGA) hosted by the EBI and the CRG under accession: EGAS00001004425. Raw sequencing files of rat data are available from GEO under accession: GSE150646.

### Ethical approval

All experiments were carried out in line with the ethical guidelines of the European Council Directive (2010/63/EU) and were approved by the local Animal Welfare and Ethics committee of the Country Commission Tübingen, Germany (§4 v. 14.11.2016).

All procedures performed using human brain tissue were in accordance with the ethical standards of the Amsterdam University Medical Center and the local Medical Ethics Committee (AMC; W15_092; W16_317) and with the 1964 Helsinki declaration and its later amendments or comparable ethical standards.

## Supplementary Material

Supplementary Figures

Supplementary Tables
