# Supplementary material for: Increased expression of myelin-associated genes in frontal cortex of SNCA overexpressing rats and Parkinson’s disease patients
Source: Aging (Albany NY). 2020 Oct 5;12(19):18889–906. doi: 10.18632/aging.103935 (PMC7732335; doi:10.18632/aging.103935)
Supplement: Supplementary Figures [file aging-12-103935-s001..pdf]

SUPPLEMENTARY FIGURES

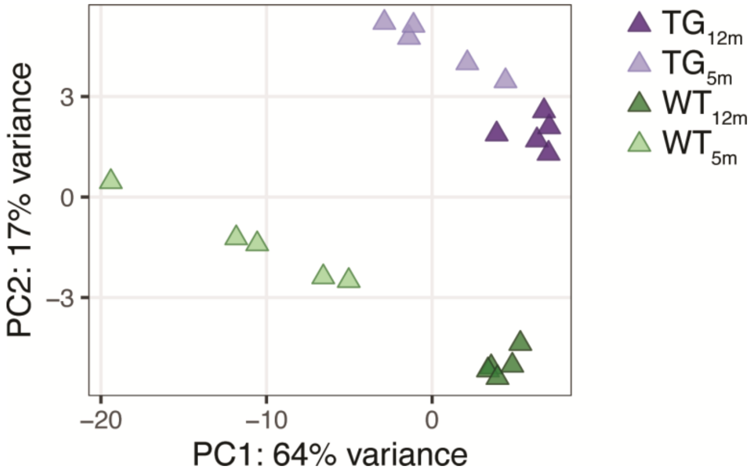

Supplementary Figure 1. Samples separate according to age and genotype. Principal component analysis of frontocortical gene expression profiles for five rat samples per experimental group. Axis percentages indicate variance contribution of the first and second principle component.

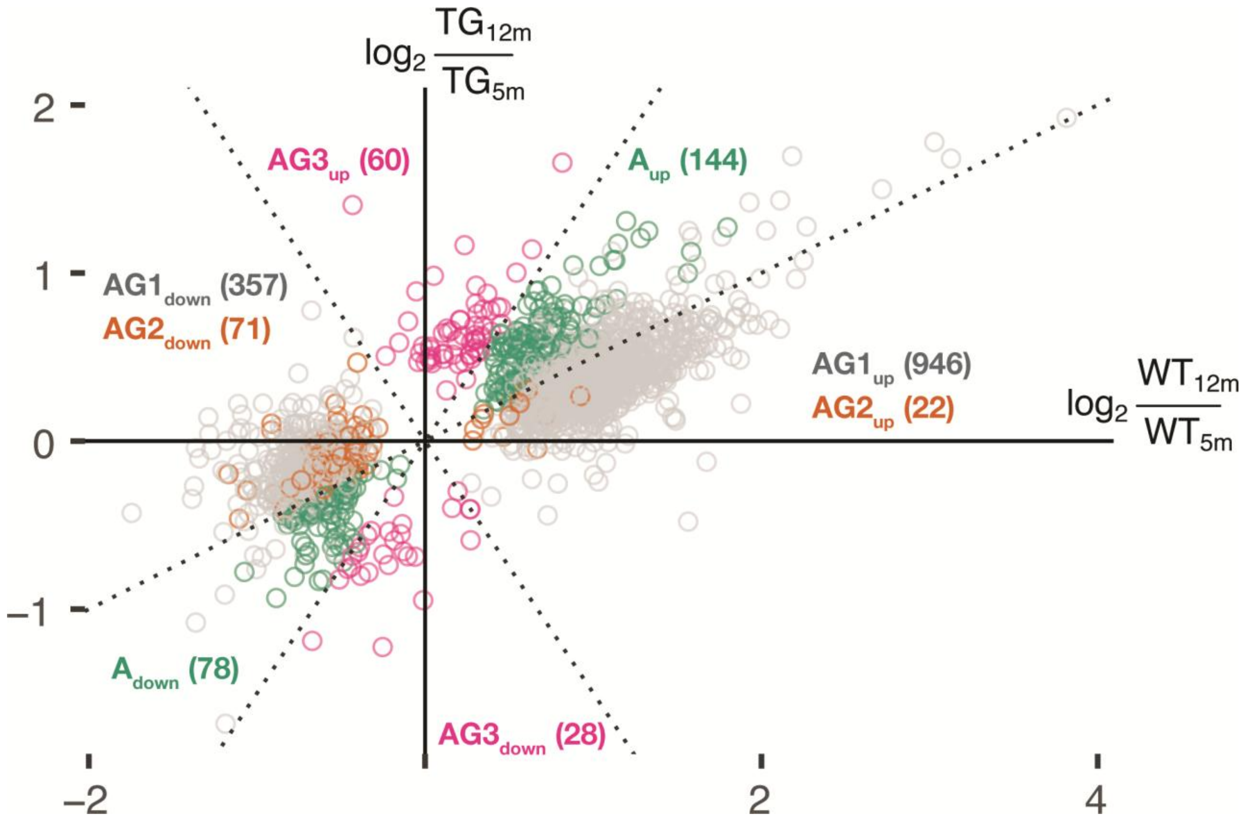

Supplementary Figure 2. Classification of DEGs based on expression patterns in 5- and 12-month-old wildtype and transgenic rats. Scatter plot of gene expression changes between 5- and 12-month-old WT (*x*-axis) and TG (*y*-axis) rats. 1706 DEGs, summarized in Figure 3A, are shown. DEGs were partitioned into four main classes based on the expression ratios of TG_{12m}/TG_{5m} (*y*-axis) versus WT_{12m}/WT_{5m} (*x*-axis) as shown. Any DEG in class A or AG2 with $log_2FC > 0.25$ in TG_{5m}/WT_{5m} was assigned to class AG1.

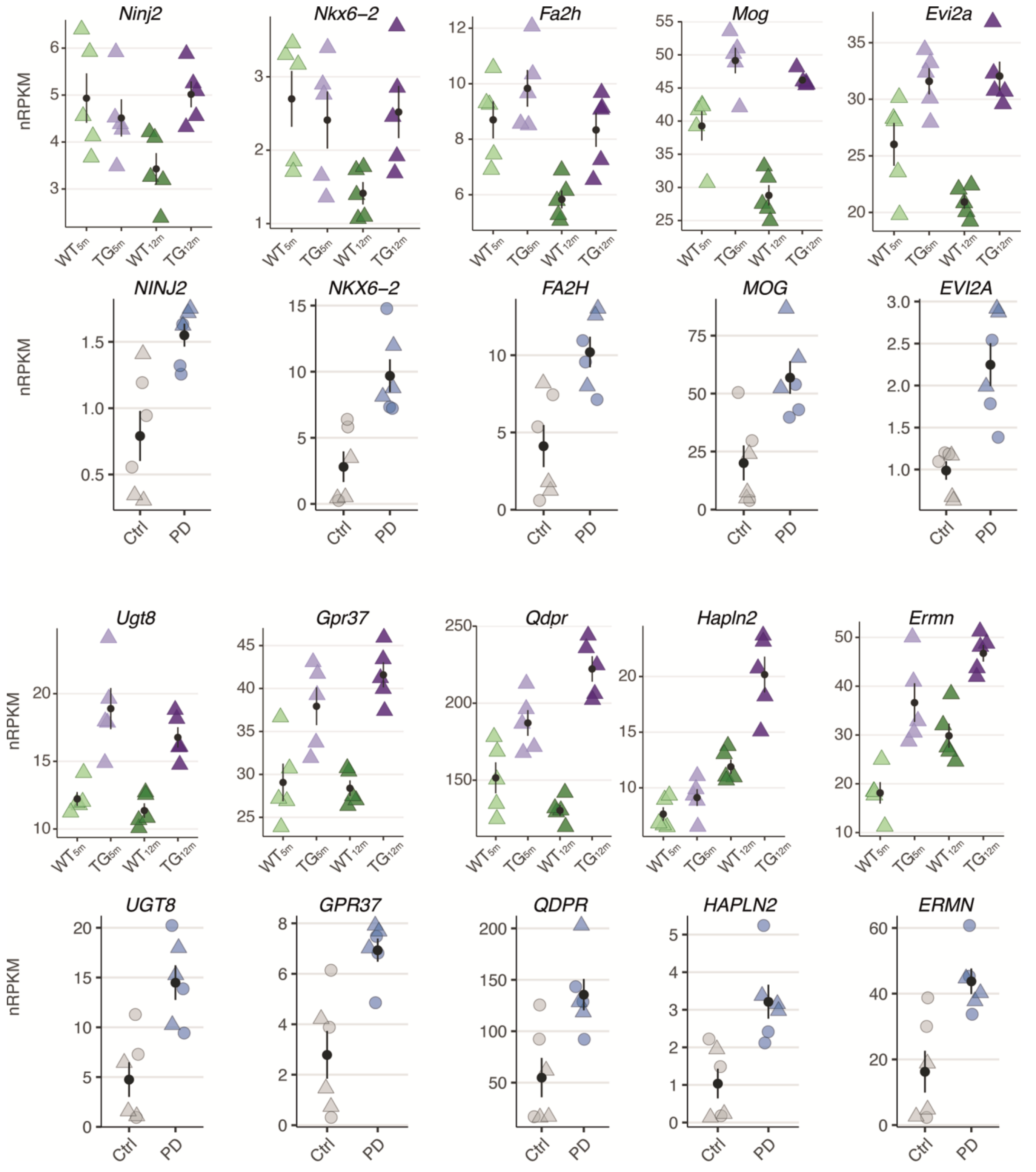

Supplementary Figure 3. Oligodendrocyte genes associated with myelination are up-regulated in frontal cortex of rats overexpressing *SNCA* and PD patients. Expression changes across experimental groups of oligodendrocyte DEGs shared between rat and human plotted as individual data points with mean ± SEM. For human data, circles represent females, rectangles males.
