# Supplementary material for: Increased expression of myelin-associated genes in frontal cortex of SNCA overexpressing rats and Parkinson’s disease patients
Source: Aging (Albany NY). 2020 Oct 5;12(19):18889–906. doi: 10.18632/aging.103935 (PMC7732335; doi:10.18632/aging.103935)
Supplement: Supplementary Tables [file aging-12-103935-s002..pdf]

SUPPLEMENTARY TABLES

Supplementary Table 1. Characteristics of human brain frontal cortex samples. Post mortem interval (PMI).

Case No.	Age, yr	Gender	PMI, hr	Clinical diagnosis	Braak α-synuclein stage	Brain region
1-014	72	female	4	PD	6	medial frontal gyrus
2-078	88	female	4	PD	6	medial frontal gyrus
3-126	83	female	4	PD	6	medial frontal gyrus
4-010	82	male	6	PD	6	medial frontal gyrus
5-029	67	male	5	PD	6	medial frontal gyrus
6-106	81	male	5	PD	6	medial frontal gyrus
C1	85	male	6	-	-	medial frontal gyrus
C2	70	male	8	-	-	medial frontal gyrus
C3	70	male	6	-	-	medial frontal gyrus
C4	82	female	6	-	-	medial frontal gyrus
C5	78	female	6	-	-	medial frontal gyrus
C6	63	female	7	-	-	medial frontal gyrus

Supplementary Table 2. List of primer sequences used for RT-qPCR.

gene	sample	sequence 5`-3`
Mag	rat	AACTGCACCCTGCTTCTCAG
	rat	ACGATGTTGGGGGTGTTGAT
Fa2h	rat	TTCTCCAAGACTGTCTGGTATAGTG
	rat	CTGGGTGAGGGTTCGGTAGT
Mog	rat	CTACTGGATCAACCCTGGCG
	rat	TCTGCACGGAGTTTTCCTCTC
Plp1	rat	CCAGTATAGGCAGTCTCTGCG
	rat	AGGTCATTTGGAACTCGGCT
Ugt8	rat	GCGAAAGGCATGGGGATCTT
	rat	GCCCTCTGCCGATAACTGG
Gpr37	rat	GTGCCCTATATCGAGGTAGCTT
	rat	TACATCTGGACGTTGGTGGC
MAG	human	GGGCTCAGTGTCATGTATGC
	human	GAATAGGGTCCGGGTTGCTC
FA2H	human	GTGTCCCCATCATCTGGGTG
	human	GCCACCGTGTACTCTGTTGTA
MOG	human	AGGGAAAGGTGACTCTCAGGA
	human	GCTCACCCAGTAGAAAGGATCTTC
PLP1	human	CTACACTGGTTTCCCTGCTCAC
	human	AAGGGGGATTTCTACGGGGG
UGT8	human	TGCTGTTGGGATAGCGAAGG
	human	TGTATGGTGGCCTCTCTCGT
GPR37	human	GTGCCCTATATAGAGGTCGCTT
	human	ACATCTGTACGTTGGTGGCA
Pgk1	rat	AGCTCCTGGAAGGTAAAGTCC
	rat	TGGCCACTAGCTGCACTAAC
Pdhb	rat	GAGTAGAGGACACGGGCAAG
	rat	CACGAACTGTCAACTGCACC
PGK1	human	GAATGGGAAGCTTTTGCCCG
	human	GCAGTGTCTCCACCACCTAT
SDHA	human	CTGTGCCTCGGTACATGGTG
	human	GGAGGGACTTTATCTCCAGGC
